# Multifunctional Actions of Ninjinyoeito, a Japanese Kampo Medicine: Accumulated Scientific Evidence Based on Experiments With Cells and Animal Models, and Clinical Studies

**DOI:** 10.3389/fnut.2018.00093

**Published:** 2018-10-08

**Authors:** Kanako Miyano, Miki Nonaka, Miaki Uzu, Kaori Ohshima, Yasuhito Uezono

**Affiliations:** ^1^Division of Cancer Pathophysiology, National Cancer Center Research Institute, Tokyo, Japan; ^2^Faculty of Pharmaceutical Sciences, Tokyo University of Science, Chiba, Japan; ^3^Division of Supportive Care Research, Exploratory Oncology Research and Clinical Trial Center, National Cancer Center, Tokyo, Japan; ^4^Innovation Center for Supportive, Palliative and Psychosocial Care, National Cancer Center Hospital, Tokyo, Japan; ^5^Department of Comprehensive Oncology, Nagasaki University Graduate School of Biomedical Sciences, Nagasaki, Japan

**Keywords:** herbal ingredient, kampo medicine, Ninjinyoeito, mixed ingredients, scientific evidence

## Abstract

Herbal medicines are currently employed for the treatment of several types of diseases, and also employed for the improvement of Quality of Life (QOL) of patients over the world, in particular, in Asian countries. In Japan, a Japanese herbal medicine namely kampo medicine has been prescribed for the improvement of QOL of patients. Ninjinyoeito (NYT), composed of 12 herbal plants, is one of kampo medicines and used for helping recovery of diseases and improving several symptoms that suffer patients such as anemia, anorexia and fatigue. Recent scientific research approaches to kampo medicines with cells and animal models enable to prove that NYT has multiple functions for improvement of symptoms. Also, clinical studies using NYT support such actions to be widely used for the improvement of symptoms that reduce the QOL of patients.

## Introduction

Ninjinyoeito (NYT) is a Japanese herbal medicine prescribed and clinically admired in Japan. NYT has multi-functional beneficial activities so that it is used for improvement of recovery from diseases or some symptoms (1–7). Further, NYT has been reported to have antiviral action on hepatitis C virus (HCV), antioxidant and immuno modulatory effects (8, 9). NYT is composed of 12 crude ingredients, including Rehmannia root, Japanese angelica root, Atractylodes rhizome, Poria sclerotium, Ginseng, Cinnamon bark, Polygala root, Peony root, Citrus unshiu peel, Astragalus root, Glycyrrhiza, and Schisandra fruit.

Recent scientific approaches to analyze the mechanism of action of kampo medicine including NYT enable to understand how kampo medicines act on patients and their functional sites of action (10–12). Further, understanding of the mechanism of action for each herbal ingredient composed of NYT help the mechanism of action of NYT. In this review, we introduce multifunctional actions of NYT by showing accumulated scientific evidence based on experiments with cells and animal models and also clinical studies using NYT.

## Actions of NYT for the improvement of diseases or symptoms

Because of multi components of NYT, NYT has a variety of sites of action so that it is widely used for improvement of several types of symptoms, as follows.

### Contribution of NYT to improve neurodisorder

NYT has been postulated to contribute of improvement of symptoms of Alzheimer's disease patients (13). In an experiment with rat embryo astrocytes, Yabe reported that NYT and its constituent onjisaponins contained in the roots of polygala tenuifolia significantly elicited nerve growth factor (NGF) secretion, a neurotrophic peptide (14). NGF is well known to be associated with the development and maintenance of cholinergic neurons in basal forebrain (15).

Sato demonstrated that the γ chain of immunoglobulin Fc receptor (FcRγ) and Fyn protein tyrosine kinase (Fyn) signaling cascade is critically involved in cuprizone-induced demyelination without any lymphocytic response with a model for certain human demyelinationing disorders (4). They showed in the model that NYT administration recovered the demyelination in cuprizone-treated mice, and also showed the site of action of NYT is the FcRγ Fyn-Rho (Rac2)—p38 mitogen-activated protein kinase phosphorylated myelin basic protein signaling, which is working in the myelination process (4).

With olfactory bulb lesions injured by Zinc sulfate perfusion, Song investigated the effects of NYT on brain monoamine and NGF (16). They showed the reductions of dopamine and its metabolites, 5-HT and its metabolite in olfactory bulb, hippocampus and substantia nigra in the model, and NYT inhibited the reduction. As a result, NYT consequently recovered the learning and memory lowered by Zinc sulfate perfusion (16).

### Myelopoiesis and erythropoiesis

Hematopoiesis is the process whereby functional, mature hematopoietic cells (red blood cells (RBCs), leukocytes, and platelets) are generated from hematopoietic stem cells in bone marrow (BM). Erythropoiesis, one aspect of hematopoiesis in which erythroid progenitors, such as burst forming unit-erythroid (BFU-B) and colony forming unit-erythroid (CFU-E) cells, is initially generated and then gives rise to erythroblasts, reticulocytes, and finally RBCs, which contain hemoglobin functioning in oxygen transport (17, 18). Failure of erythropoiesis results in a shortage of or damage to RBCs and underlies anemia. NYT has been reported to overcome anemia resulting from anticancer therapies in humans (1). In mice treated with an anticancer drug 5-fluorouracil (5-FU) and received anemia due to 5-FU, oral administration of NYT protected against hematotoxicity and induced immature erythroid progenitor cells. Also, oral administration of NYT improved 5-FU-induced anemic conditions, determined by measurement of reticulocyte and RBC numbers, hemoglobin and hematocrit levels in peripheral blood, and increases in BFU-E and CFU-E in BM in mice (3).

### Modulation of immunoresponses

Takaku reported that NYT enhanced synergistically the antitumor vaccine effects induced by CD8^+^ T cells. They demonstrated that NYT is a useful adjuvant herbal medicine for cancer immunotherapy (6).

Multiple myeloma (MM) is an incurable malignancy of plasma cells and includes the involvement of chemokines, cytokines and growth factors secreted from MM (19). For these patients over 65 years, Melphalan-prednisone (MP) therapy has been employed (5). In this study, MP treatment lowered the concentrations of several cytokines such as RANTES, sE-selectin, but not Ang-2 and VEGF. When treated together with NYT, it enhanced MP-associated reduction and also reduced Ang-2 and VEGF. During this combination therapy, immunoglobulin concentrations were significantly improved and improved general fatigue of patients, suggesting beneficial immunotherapeutic effects of NYT on MM patients (5).

### Neuropathic pain

NYT has been reported to have anticytotoxic activities. In neuron-like pheochromocytoma PC12 cells, an antitumor drug oxaliplatin induced neurodegeneration. With this model, NYT was found to prevent the neurodegenerative effects by oxaliplatin (20). Further, in mice model, the same authors reported that NYT and its gradient ginseng, in particular, ginsenoside Rg_3_, suppressed oxaliplatin-induced neurite damage and neuropathic pain (7).

### Scavenging action of NYT on several types of free radicals

NYT has been reported to have scavenging activity of several types of radicals including 1,1-diphenyl-2-picrylhydrazyl (DPPH) radicals in *in vitro* system as well as ascorbic acid and α-tocopherol (8). They proposed that examination of radical scavenging ability of kampo medicines is a better method for evaluating the efficacy of kampo medicines (8). Actually, Hange-shasinto, a kampo medicine that is widely prescribed for therapeutic use of oral stomatitis, has been reported to have radical scavenging effects in six out of seven herbal ingredients consisting of Hange-shasinto (21).

### Frailty in locomotor disease

Frailty is a syndrome that includes broad problems of senility and consists of three domains: physical, psychological, and social. Kampo medicines are used for intervention in cases of hypofunction in a mental or physical state (22). For frailty, NYT is useful in patients with symptoms of coldness or cutaneous dryness. NYT demonstrates hematopoietic activity and is effective for osteoporosis management (22). Also, NYT was effective for anorexia of aging in Alzheimer's disease (23).

## Accumulated evidence of the effects of each herbal plants consisting of NYT

Here we show the effects of each herbal ingredient that consist of NYT, as follows (Table 1).

**Table 1:** Physiological functions of basic components from each ingredients in NYT.

	**Herbal ingredients**	**Formula in NYT**	**Basic components**	**Physiological functions**	**References**
1	Rehmannia Root	4.0 g	catalpol	• Antiosteoporotic • Antineurodegenerative • Regulation of Na^+^/Ca^2+^ exchanger 3	(24–27)
2	Japanese Angelica Root	4.0 g	ligustilide	• Antiinflammatory	(28–30)
3	Atractylodes Rhizome	4.0 g	atractylenolides	• Improve symptom of cancer patients	(31)
4	Poria Sclerotium	4.0 g	pachymic acid	• Antitumor • Inhibition of enzymes from acyl ghrelin (active) to des-acyl ghrelin (inactive)	(32–37)
5	Ginseng	3.0 g	ginsenoside	• Antitumor • Antiinflammatory • Antioxidative	(7, 38–41)
6	Cinnamon Bark	2.5 g	cinnamaldehyde	• Antiinflammatory • Antioxidative • Antitumor • Neuroprotective	(42–46)
7	Polygala Root	2.0 g	tenuigenin	• Neuroprotective • Antiinflammatory	(47–49)
8	Peony Root	2.0 g	paeoniflorin	• Pain relief • Ca^2+^ channel inhibition	(50–52)
9	Citrus Unshiu Peel	2.0 g	hesperidine, hesperetin	• Neuroprotective • Antioxidant • Antiinflammatory	(53–56)
10	Astragalus Root	1.5 g	astragaloside, isoastragaloside	• Elevation of adiponectin production • Antitumor	(57–60)
11	Glycyrrhiza	1.0 g	glycyrrhizin, glycycoumarin	• Antiinflammatory • Antioxidative • Neuroparotective • Keep ghrelin levels as pachimic acid	(32, 61, 62)
12	Schisandra Fruit	1.0 g	schizandrin	• Antiinflammatory • Enhancement of skeletal muscle endurance	(63–65)

### Rehmannia root

Rehmannia root has been reported to have antiosteoporotic and neuroprotective effects (25, 26). Catalpol, the main component in Rehmannia root, inhibited ischemia-induced promyelinating oligodendrocyte damage by the regulation of intracellular homeostasis through Na^+^/Ca^+^ exchanger 3 activity (27). Also, catalpol reduced insulin resistance caused by high-fat diet and inflammation of adipose tissues by the inhibition of the c-Jun N-terminal kinase and nuclear factor-κ B-induced signaling (24). Catalpol is presented as a potential therapeutic for neurodegenerative diseases (25).

### Japanese angelica root

Japanese angelica root contains many of bioactive components. One of main components is ligustilide (30). Ligustilide has antiinflammatory and antinociceptive effects (28, 29). Qian reported that ligustilide ameliorated pain caused by inflammation and inhibited TLR4 upregulation in spinal astrocytes induced by injection of complete Freund's adjuvant (30).

### Atractylodes rhizome

For atractylenolide I (ATR) in Atractylodes rhizome, a pilot randomized study (*n* = 11) of ATR on cachexia patients with gastric cancer showed ATR was significantly more effective than fish-oil-enriched nutritional supplementation in improving appetite and Karnofski performance status, suggesting ATR might be beneficial in alleviating cachexic symptoms in gastric cancer patients (31).

### Poria sclerotium

Poria sclerotium has a long history as a herbal medicine and a wild spectrum of pharmacological activities such as antitumor, antioxidant, antiinflammatory effects (33). Pachymic acid, one of the main components of Poria sclerotium, has been reported to have antitumor effects to several types of tumor cells (34–37). On the other hand, Rikkunshito, a kampo medicine prescribed for gastrointestinal disorders, enhances orexigenic ghrelin-mediated signaling (10, 12). Acyl ghrelin is the active hormone and it degrades inactive form of des-acyl ghrelin by ghrelin deacylating enzymes (32). Pachymic acid in Poria sclerotium as well as glycycoumarin in Glycyrrhiza and 10-gingerol in Ginger inhibited this enzyme activity and kept circulating acyl ghrelin levels, thus maintaining the effect of ghrelin (32). NYT contains both Poria sclerotium and Glycyrrhiza, suggesting these two ingredients may involve the ghrelin-mediated signaling in NYT (12).

### Ginseng

Ginseng has a wide range of pharmacological activities including antitumor, antiinflammatory, antioxidative and inhibition of cardiovascular diseases (40, 41). Further, the extract of Ginseng (Rg_3_) showed a protective effect against neurite damage induced by oxaliplatin (7). Ginseng extracts partially relieved oxaliplatin-induced neuropathic pain (7). In the effect to central neurons system, many of studies suggest ginseng causes improvement in memory and learning in the aged or damaged brain in rats (38). Also, ginsenoside-Rg_2_ from Panax ginseng protected memory impairment through antiapoptosis with a rat model having vascular dementia (39).

### Cinnamon bark

Cinnamon bark is widely used in food and traditional herbal medicine including NYT. Cinnamon has been reported to elicit diverse biological functions such as antiinflammatory (42) antioxidant (43), antimicrobial (43), and antitumor activity (44). A recent report showed that cinnamaldehyde has potent neuroprotective effects against oxidative stress and apoptosis induced by glutamate with rat pheochromocytoma PC12 cell as a neuron model (45). As for antioxidative effect of cinnamon bark, Sedighi recently reported that the extract of cinnamon bark with ethanol could protect the heart injured by ischemia–reperfusion probably because of its antioxidant properties (46).

### Polygala root

Tenuigenin in Polygala root has been reported to have neuroprotective effects. For example, it promoted hippocampal neural stem cell proliferation and differentiation (48) and it also has protective effects in cultured hippocampal neurons (47). Furthermore, tenuigenin has antiinflammatory effects and it inhibited lipopolysaccharide-induced inflammatory responses in microglia via activating the Nuclear factor E2-related factor 2-mediated heme oxygenase 1 signaling pathway (49).

### Peony root

Products from Peony root have been used for pain relief (52) and antispasmodic (51). Paeoniflorin, a glycoside isolated from Peony root, is reported to inhibit L-type Ca^2+^ currents in neuroblastoma NG108-15 cells (50). These effects may partially explain paeoniflorin or Peony root to cause neuronal or neuroendocrine inhibitory effects as well as pain relief (50).

### Citrus unshiu peel

A flavonoid hesperetin in Citrus unshiu peel is reported to have a variety of biological activities, including anticancer, antiviral, antioxidant, neuroprotective and antiinflammatory properties (55). For example, hesperidin ameliorates cognitive dysfunction, oxidative stress and apoptosis against aluminum chloride induced rat model of Alzheimer's disease (56). Administration of citrus unshiu peel is reported to reverse age-induced demyelination (54). Also, restoration of FcRγ/Fyn signaling activated by citrus unshiu peel repaired central nervous system demyelination (53).

### Astragalus root

Main components in Astragalus root are astragaloside and isoastragaloside. Xu reported that astragaloside and isoastragaloside in Astragalus root by screening of 50 medical herbs elevated circulating adiponectin levels by enhancing adiponectin production in mouse adipocytes (60), a well-known hormone to reduce risk of obesity, cardiovascular, and diabetic pathophysiology (57, 58). Also, Astragalus extract was reported to inhibit destruction of gastric cancer cells to methothelial cells by antiapoptosis, suggesting that Astragalus root can be used an adjuvant chemotherapeutic agent in gastric cancer therapy (59).

### Glycyrrhiza

Glycyrrhiza extracts such as glycyrrhizin has been reported to have a variety of biological activities such as antiinflammation, anticancer, antioxidative, antiviral, antimicrobial effects as well as neuroprotective and immunomodulatory effects (61, 62). Also, as mentioned in the above section of Poria sclerotium, glycycoumarin in Glycyrrhiza inhibited circulating deacylating enzymes to consequently enhance the ghrelin-mediated signaling (32).

### Schisandra fruit

Schisandra fruit has many of bioactive activities. Kim reported that its extracts improved endurance and energy metabolism by upregulation of proliferator-activated receptor γ coactivator-1α (PGC-1α) in the skeletal muscle of exercised rats (64). Moon reported schizandrin, a main component of Schisandra fruit had antiinflammatory effects and it could be useful for the inflammatory and atopic diseases (63). Further, schizandrin B, an extract from Schisandra fruit ameliorated the lipopolysaccharide-induced depressive-like behaviors by attenuation of inflammation in the hypothalamic paraventricular nucleus and central nucleus of the amygdala in mice (65).

## Clinical studies using NYT

Although not so many, clinical studies regarding the effects of NYT on healthy human or patients have been conducted. Clinical reports showed that BT-11, the extract of dried roots of Polygala that is ingredient of NYT, had memory-enhancing effects in a randomized, placebo-controlled and double-blind study of BT-11 in healthy adults (66). The extract is also reported to have enhancing effects in cognition in elderly humans in a randomized, placebo-controlled and double-blind study (67).

In clinical study with Alzheimer's patients (23 patients) over 2-year period, two groups between donepezil (11 patients) and donepezil with NYT (12 patients) treatment were compared and assessed. Tests performed included Mini-Mental State Examination and the Alzheimer's Disease Assessment Scale-cognitive component-Japanese version for cognitive function (13). Further, the Neuropsychiatric Inventory was used to evaluate the patients' mood status at baseline and every 6 months for 2 years (13). Authors demonstrated that A 2-year follow-up of patients receiving donepezil with NYT treatment showed an improved cognitive outcome and alleviation of Alzheimer's disease-related depression, judged from several tests mentioned (13). Further, an open-label pilot study with frail Alzheimer's disease patients showed that NYT could be a new-type dementia therapeutic agent with low risk of adverse effects, which improves anorexia, apathy, and cognitive dysfunction (23).

Nowadays, complementary and alternative medicine such as traditional Chinese medicines and Japanese kampo medicines is frequently used together with western medicines for treatment of diseases including chronic kidney diseases (68). In an open-label clinical study, Hsiao reported that NYT could decrease chronic inflammation and increase QOL in 59 (27 NYT and 32 control) patients treated with hemodialysis due to chronic kidney diseases (68). In addition, a randomized controlled trial with NYT was conducted in patients receiving ribavirin, which shows a strong antiviral effect on hepatitis C. Ribavirin is known to cause serve anemia and this is the major problem. In the study, NYT was shown to ameliorate the anemia induced by ribavirin in patients with hepatitis C (1). Also, combination of a clinical study with hepatitis C patients and *in vitro* studies have shown that NYT and some of its ingredients were effective in the treatment of chronic hepatitis C (69). With regard to hepatocellular carcinoma, one study in cancer patients having advanced hepatocellular carcinoma, together with experimental study with a rat hepatocellular carcinoma model revealed that NYT could enable continuous administration of the anticancer drug sorafenib due to suppression of failure of liver functions and reduction of platelets (70). Lastly, a clinical I/II open-label study with NYT showed that NYT was safe and useful for improvement of fatigue in nonanemic cancer survivors (71).

## Conclusion

Since NYT is consist of 12 herbal ingredients and each ingredient has potent improving activities of a variety of symptoms in patients with significant biological activities, the beneficial effect of NYT could be promising. Accordingly, not a few of clinical studies with NYT are now ongoing. In the near future we will recognize and understand the beneficial effects of NYT to patients suffering from many of unpleasant symptoms, owing to the progression of evidence-based scientific researches with NYT.

## Author contributions

In this review, KM and YU mainly wrote and summarized the data of NYT. MN, MU and KO contributed to summarize the effects of each ingredient composed of NYT. As a results, all authors have made a substantial, direct and intellectual contribution to the work.

### Conflict of interest statement

The authors declare that the research was conducted in the absence of any commercial or financial relationships that could be construed as a potential conflict of interest.
